# Intonation processing of interrogative words in Mandarin: an event-related potential study

**DOI:** 10.3389/fnhum.2023.1326602

**Published:** 2023-12-15

**Authors:** Rui Wang, Mengru Wang, Georgi V. Georgiev

**Affiliations:** ^1^School of Educational Science and Technology, Anshan Normal University, Anshan, Liaoning, China; ^2^Center for Ubiquitous Computing, University of Oulu, Oulu, Finland

**Keywords:** interrogative words, mismatch negativity, intonation, Mandarin tone, language lexical semantic function, acoustic features, event-related potential

## Abstract

Intonation is the variation in pitch used in speech, which forms the premise of tonal and non-tonal languages. Interrogative words are words that introduce questions. Previous research lacks clarity regarding the specific cues used in the processing of word intonation. To address this gap, this study used the event-related potential electroencephalogram (EEG) research method to explore the intonation processing of tone two (mid-rising) interrogative words in Mandarin. For this, the word “shui,” meaning “who,” was selected as the experimental material. To avoid the influence of the environment, gender, and semantics, the Hum version, corresponding to the stimulus material, was also adopted for the experiment. This study used a passive oddball paradigm to examine the clues of intonation information processing in automatic cognitive processing through amplitude, latency, time window, and evoked location potential mismatch negativity. The standard stimulus was the declarative intonation with a high probability of occurrence (90%), and the deviant stimulus was the interrogative intonation with a low probability of occurrence (10%). In the time window of 370–450 ms, the mismatch negativity was found at the F3, F4, C3, Cz, and C4 channels. The findings show that, in the passive oddball paradigm, lexical semantics are essential for intonation processing at the pre-attentive level, which is dominated by the frontal and central areas of the brain. The results support the functional and comprehensive hypotheses that the processing of intonation is based on the function of language and that bilateral regions are involved in this processing. This study makes an important contribution by providing event-related potential evidence that lexical semantics plays a key role in the pre-attentive processing of intonation, as shown by the significant differences between semantic and non-semantic conditions.

## 1 Introduction

Intonation is common information in intonation and non-intonation languages, and it is also the mode of pitch change at the sentence level. The change in intonation is mainly formed through a change in the overall pattern of pitch, and its realization is a comprehensive effect that includes coordinated changes in pitch, length, and intensity (Yang, 2015). Mandarin Chinese tones can be described phonetically as having high-level (tone 1), mid-rising (tone 2), low-rising (tone 3), or high-falling (tone 4) pitch patterns. As there are different degrees of similarity among the four tones in terms of acoustic characteristics, these differences could influence pre-attentional processing (Xu, 1997). Interrogative words refer to words that can help raise questions, and their basic usage is to express inquiries and access unknown information (Xue, 2014).

Previous research lacks clarity regarding the specific cues used in the processing of word intonation. This study examined automatic intonation processing using a passive oddball paradigm, focusing on the amplitude, latency, time window, evoked location, and other mismatch negativity (MMN) information. This study hypothesized that lexical semantic function is important during early intonation processing. The acoustic hypothesis is supported if the semantic and non-semantic conditions are not distinguishable.

## 2 Background

### 2.1 Brain lateralization in speech processing

Lateralization of the brain during speech information processing, which is also known as asymmetry, has attracted considerable attention from researchers. Researchers generally believe that information processing in language, regardless of speech perception or production, is a function of language. Both aspects rely primarily on the left hemisphere of the brain (Markus and Boland, 1992). Studies suggest that intonation processing involves brain areas related to speech and premotor functions as well as universal auditory mechanisms, and it shares similarities across languages, but with some dissociations for tonal language speakers (Chien et al., 2020). Intonation processing among tonal-language speakers involves increased frontotemporal connectivity, thereby suggesting the involvement of a phonological network (Chien et al., 2020). Overall, intonation processing involves the activation of specific brain regions, including the left inferior frontal gyrus and the bilateral temporal regions, as well as the establishment of functional connectivity within phonological networks.

However, in the processing of speech prosodic information, there is a phenomenon of the lateralization of brain functions, whereby the information carried in prosodic information has different performances in a specific time span, and there are differences in the brain regions that integrate such information. Regarding the processing of speech prosodic information, previous studies have mainly proposed four viewpoints: the functional hypothesis (Liberman and Whalen, 2000), the acoustic hypothesis (Zatorre et al., 2002), the comprehensive hypothesis (Gandour et al., 2004), and the two-stage model (Luo et al., 2006). In previous studies, the acoustic (Tong et al., 2005; Ren et al., 2009) and functional hypotheses (Gandour et al., 2004; Wong et al., 2005) have been supported through many experiments. However, most of the studies focus on the level of lexical recognition and lexical-tonal information processing, and they rarely discuss the brain mechanisms behind intonation processing.

The functional hypothesis, which is also known as the task-dependent hypothesis, states that pitch processing is biased toward the left hemisphere of the brain when pitch patterns carry more speech information. When less verbal information is conveyed in pitch patterns, pitch processing is biased toward the right hemisphere of the brain (Liberman and Whalen, 2000). Gandour et al. (2002) used Thai vocabulary to compile experimental materials, and they employed functional magnetic resonance technology (fMRI) to study the processing of Thai sounds and Thai vowel lengths by selecting native Chinese and Thai speakers under both phonetic and non-phonetic conditions. The brain mechanisms of the two groups of participants in processing spectral information and processing time information related to language were investigated. The results showed that only the native Thai speakers experienced activation in the left sub-prefrontal cortex of the brain under the condition of tone judgment. Gandour et al. (2003) conducted a cross-language study using functional magnetic resonance imaging (fMRI) and found that when processing information related to the lexical tone of Chinese words, native Chinese speakers mainly relied on the left hemisphere for information processing, whereas the right hemisphere was mainly relied on when processing information related to intonation.

The sound hypothesis, which is also known as the cue-dependent hypothesis, states (Zatorre et al., 2002) that the acoustic structure of auditory stimuli determines the functional lateralization of both hemispheres of the brain. The sounds that reflect spectral changes are mainly processed in the right hemisphere of the brain, whereas the sounds that reflect temporal changes are mainly processed in the left hemisphere. Tong et al. (2005) used fMRI to investigate the neural mechanisms of native Chinese and English speakers when processing prosodic information in Mandarin Chinese. The study found that both groups showed right-sided shifts in the medial frontal gyrus of the brain. Activation in the left superior limbic gyrus and posterior middle temporal gyrus among native Chinese participants was not observed among native English participants. A change in pitch pattern can lead to changes in both lexical tone and lexical intonation. In a study on the neural mechanism of tone, to investigate the influence of language background on automatic processing, Kaan et al. (2007) adopted the passive oddball paradigm and selected three groups of participants whose mother tongues were Chinese, English, and Thai, respectively, to study the tonal processing of Thai. The results showed that Chinese and English participants performed similarly in the discrimination of Thai low and mid tones. Under these two experimental conditions, the discrimination of Thai tones induced MMN among the three groups of participants, and the mean amplitude of MMN was not significantly different. Ren et al. (2009) studied the neural mechanisms behind the automatic processing of pitch information and found that regardless of whether the change in pitch caused a change in the intonation of words with linguistic function or words without linguistic function, the participants showed a processing advantage in the right side of the brain regardless of whether the change existed in the linguistic or non-linguistic environments. Subsequently, Ren et al. (2011) explored the brain mechanisms behind Chinese intonation automatization and found that the processing of Chinese tone-two intonation in the absence of semantics can produce a processing advantage in the right hemisphere, independent of the integration of time windows.

According to the comprehensive hypothesis by Gandour et al. (2004), the recognition of speech prosody is mainly modulated by the right hemisphere of the brain, responsible for complex speech analysis; however, this recognition is mainly performed by the left hemisphere of the brain, responsible for language processing. This was reported in a study by Schmithorst et al. (2006), who used an fMRI to study prosodic information processing in 5-year-old children. In their study, children were asked to complete an experimental task of prosodic matching. The results suggested that the children had a right hemispheric advantage in processing prosodic information, but both sides of their brain networks were activated. Subsequently, Wartenburger et al. (2007) studied the speech content and prosody processing of 4-year-old children using the research method of near-infrared spectroscopy. The results demonstrated that the right frontal and temporal lobes of the brain showed significant activation when only the prosodic information of speech was processed, whereas the left hemisphere showed greater activation when the content of speech was processed.

According to the two-stage model (Luo et al., 2006), speech is initially processed in the pre-attention stage as a general sound signal rather than a function-specific signal, after which it is mapped into a semantic representation through the activation of neural circuits. Luo et al. (2006) proposed that the brain relies mainly on acoustic cues during early word processing, which is consistent with the sound hypothesis. The hemispherical advantage of speech processing depends on the acoustic structure of the first stage, i.e., the need to solve the computational problems posed by the precise extraction of time and spectral information, but at the same time, it depends on the linguistic function of the second stage, i.e., the need to integrate information in the language. This theory suggests that the acoustic and functional hypotheses are not mutually exclusive but simply work at different stages of auditory processing.

### 2.2 Mismatch negativity

Mismatch negativity (MMN) was first discovered by Näätänen et al. (1978). The most classic paradigm for inducing MMN is the oddball paradigm of binaural listening. The standard stimulus with a higher probability and the deviant stimulus with a lower probability are presented to the participants through the left and right ears, respectively, during the experiment, and the participants are required to pay attention to the sound in one ear while ignoring the sound in the other ear. It was found that whether the deviant stimulus appeared in the attentive or non-attentive ear, it caused a larger negative wave than the standard stimulus. MMN can present a novel time window in auditory processing that reflects the neural mechanisms behind brain processing, thereby providing a new understanding of the brain processes that form the biological basis of central auditory perception, different forms of auditory memory, and attentional processes that control auditory sensory input into conscious perception and higher forms of memory (Näätänen et al., 2007). MMN usually reaches its peak 150–250 ms after a stimulus, and its latency is shortened with an increase in the amplitude induced by a stimulus (Näätänen et al., 2007; Winkler, 2007).

In previous studies, it was unclear which cues were used to process the intonation of words. By comparing the main hypotheses of prosodic information processing, it is not difficult to find that the proponents of the functional and acoustic hypotheses use different research methods. The research advantage of functional magnetic resonance imaging (fMRI) is that it has a strong spatial positioning function but at the expense of time resolution. Therefore, studies on the automatic processing of intonation information cannot be conducted using fMRI. Ren et al. (2011) used the passive oddball paradigm to explore intonation information processing. They found that the intonation information processing of Mandarin tone two (mid-rising) words did not trigger MMN under the condition of semantic meaning. Mandarin tone two (mid-rising) is a special tone with a rising pitch contour that is very similar to interrogative intonation. The differences between declarative and interrogative intonation in Chinese showed that interrogative intonation has a higher phrase curve than declarative intonation (Yuan et al., 2002).

In this study, the Event-related Potential (ERP) research method was used to explore the intonation processing of interrogative words in Mandarin tone two (mid-rising). The tone-two interrogative word “shui” (which means “who” “谁”) was selected as the experimental material (semantic condition). Meanwhile, to avoid the influence of the environment, gender, or semantics, the Hum version corresponding to the stimulus material was also adopted for the experiment (non-semantic condition). In this study, a passive oddball paradigm was used to examine the clues of intonation information processing in automatic processing through amplitude, latency, time window, evoked location, and other MMN information. This study proposed the following hypotheses: if lexical semantic function is important during the early stage of intonation processing, the results support the function hypothesis, and if there is no difference between semantic and non-semantic conditions, the results support the acoustic hypothesis.

## 3 Materials and methods

### 3.1 Participants

Seventeen undergraduates and postgraduates from Liaoning Normal University, including 11 males and six females, were recruited to participate in the experiment. The participants’ age ranged from 20 to 26 years old, with an average age of 23 years old. The participants were right-handed native speakers of Mandarin Chinese; had normal hearing, vision, or corrected vision; had no neurological diseases or internal or external brain damage; and did not use addictive drugs continuously. Before the formal experiment began, each participant was informed of the specific experimental processes, read and signed the informed consent form, and received a reward afterward.

### 3.2 Experimental design

A single-factor, two-level (semantic and non-semantic), within-subject experimental design was adopted. In the semantic condition, the sound material was the tone two interrogative word “shui” (meaning “who” “谁”). In the non-semantic condition, the sound material was a “Hum” version of the tone-two interrogative word “shui” (meaning “who” “谁”).

### 3.3 Materials

This study comprised two experiment conditions, in which the stimulus sequence, composed of sound stimuli, included two types of stimuli: (i) a standard stimulus with a high probability of occurrence (90%), and (ii) a deviant stimulus with a low probability of occurrence (10%). The first condition comprised the tone two interrogative word with semantic meaning, in which the standard stimulus was the declarative intonation of “shui,” and the deviant stimulus was the interrogative intonation of “shui.” The second condition comprised the tone two interrogative word with non-semantic meaning, in which the standard stimulus was the declarative intonation of “shui” in the Hum version (to avoid the influence of the environment, gender, and semantics), and the deviant stimulus was the interrogative intonation of “shui” in the Hum version.

**FIGURE 1:**
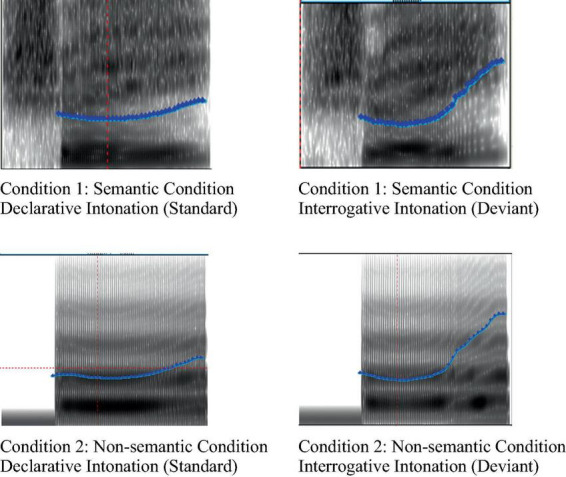
Spectrum diagram of sound stimuli.

All the sound stimuli used in the experiments were recorded by professionals (Mandarin Level 1 B), using a CSL-4500 voice workstation. The frequency parameters of all sound stimuli were set at 44100 Hz. After recording each sound more than three times, the best sound was selected as the official experimental material. All sounds were processed using Praat software^1^ (Styler, 2013). Among these, the duration of the stimulus in both intonations (declarative and interrogative) of the tone-two interrogative word “shui” was 510 ms. The sound stimuli used in both conditions had the same acoustic parameters. The start time of the sound was the same for both the standard and deviation stimuli. The average and maximum amplitudes of the sound for both stimuli were also adjusted to be the same using Praat software. The spectrum diagram of the stimulus is shown in Figure 1, where the ordinate is the fundamental frequency (f0) of material, and the abscissa is the time (ms). Before the formal experiment started, ten students, who did not participate in the formal experiment, evaluated the stimuli used in the experiment.

### 3.4 Procedure

The experiment consisted of two different conditions (blocks), each containing 715 sound stimuli (trials) with a time interval (offset-to-onset ISI) of 700 ms. The first 15 sound stimuli in each condition were not included in the EEG recording or statistical analysis. The probability of the standard stimulus was 90% (630 times) and the probability of the deviant stimulus was 10% (70 times). In each stimulus sequence, the order of stimulus presentation was processed pseudo-randomly in advance to ensure that there were at least two standard stimuli between each deviant stimulus presentation. The order of presentation of the experimental conditions was divided into two groups that were balanced between the participants. In the experiment, the sound stimulus was presented to the participants through headphones, and the sound volume was uniformly set to 70 dB. In this experiment, the participants were asked to choose a silent movie according to their own preferences and were highly deprived of attention. They were then asked to ignore the sound in the headset during the formal experiment and focus on watching the selected silent movie.

The experiments were conducted in a soundproof and lightproof laboratory with soft lighting. Each participant sat on a chair 65 cm away from the computer monitor. According to the measured head circumference of the participants, the test chose the appropriate electrode cap to wear, and it adjusted the resistance of each electrode cap, as long as the resistance in each electrode was reduced to less than 50 KΩ. After the electrodes were adjusted, the following instructions were presented to the participants through a computer monitor: “In the subsequent experiment, you will hear a series of sounds through the headset while watching a silent movie. Please ignore the sounds in the headset and watch the movie carefully. During each break, I will ask you about the movie plots. Please try your best to answer these questions. Please try your best to control your body movements and blink your eyes during the experiments. If you confirm your participation, press any key to access the experiment.” The participants were asked to read the instructions carefully and confirm their understanding before starting the experiment. After completing the practice experiment, the participants entered the formal experiment.

**FIGURE 2:**
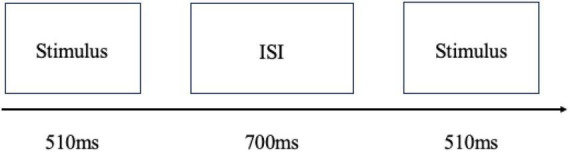
Experimental sequence. ISI 700 ms = offset-to-onset stimulus interval.

In the formal experiment, the sound stimulus used was presented to the participants through headphones. At the beginning of each experimental condition, 15 standard stimuli were presented successively. After the end of one sound stimulus, there would be a 700 ms sound interval, after which the next sound stimulus would be presented successively, and the sounds would be presented in good pseudo-random order in front of the event. After all 715 sounds were presented, the experiment was completed. After each experimental condition, the participants rested for a certain amount of time. The duration of rest was based on personal preferences. The specific presentation process of the experimental stimuli is illustrated in Figure 2.

### 3.5 EEG recordings

**FIGURE 3:**
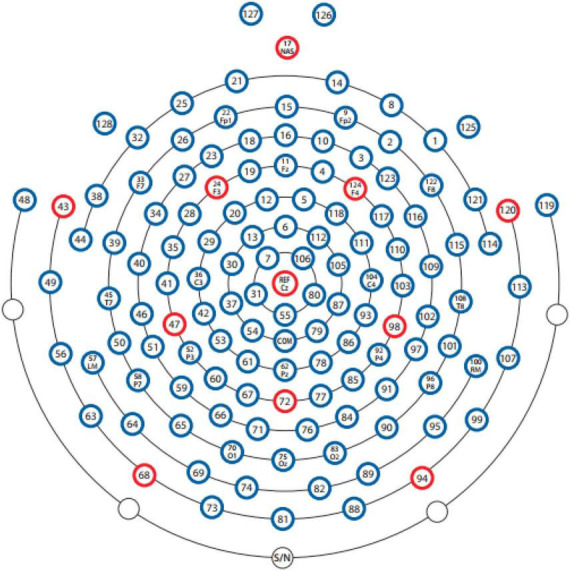
Electroencephalogram (EEG) channel distribution.

The Electroencephalogram (EEG) equipment produced by EGI (Electrical Geodesic, Inc.)^2^ was used to collect brain signals. A 128-channel electrode cap, 300-fold amplifier, and corresponding Electroencephalogram (EEG) system (Net Station 5.0) were used (Figure 3). In the experiment, the electrode distribution of the electrode cap was arranged according to the 10–20 system in the international standard, and the Cz point was selected as the reference electrode in the original record. The 128 electrodes for recording Vertical Electrooculography (VEOG) and Horizontal Electrooculography (HEOG) were included in the electrode cap, and the electrodes were adjusted to the standard recording position for data acquisition before the experiment. The sampling rate of the EEG device was 250 Hz, and the resistance of each electrode of the electrode cap was reduced to less than 50 kω throughout the experiment.

### 3.6 EEG data analysis

All recorded Electroencephalogram (EEG) data were analyzed using the MATLAB software (MATLAB 2022b, MathWorks, Inc.). The EEGLAB toolbox (EEGLAB v. 2023.0) (Delorme and Makeig, 2004) and customized scripts were used to perform the preprocessing analysis. During preprocessing, the continuous data were filtered through 0.1 Hz high pass filtering and 30 Hz low pass filtering. The filtered data were segmented according to the markers, and the epoch length was 1100 ms, including a baseline of 100 ms. Head motion artifacts were recognized and removed manually, and the EEGLAB independent component analysis (runICA) function was used to correct the electrooculogram (EOG) artifacts. Thereafter, automatic detection was used to remove the whole epoch containing the poor EEG portions with a wavelet greater than 100 μV. The data were referenced to the bilateral mastoid electrodes (E57 and E100). The MMN waveform was obtained by subtracting the standard stimulus from the deviant stimulus. The time window of MMN was determined to be 370 ms–450 ms according to the peaks in the total average amplitude of the ERP generated in the experiment. Nine electrodes (F3, C3, P3, F4, C4, P4, Fz, Cz, and Pz) were selected for the statistical analysis.

## 4 Results

### 4.1 Analysis of mean amplitude

**FIGURE 4:**
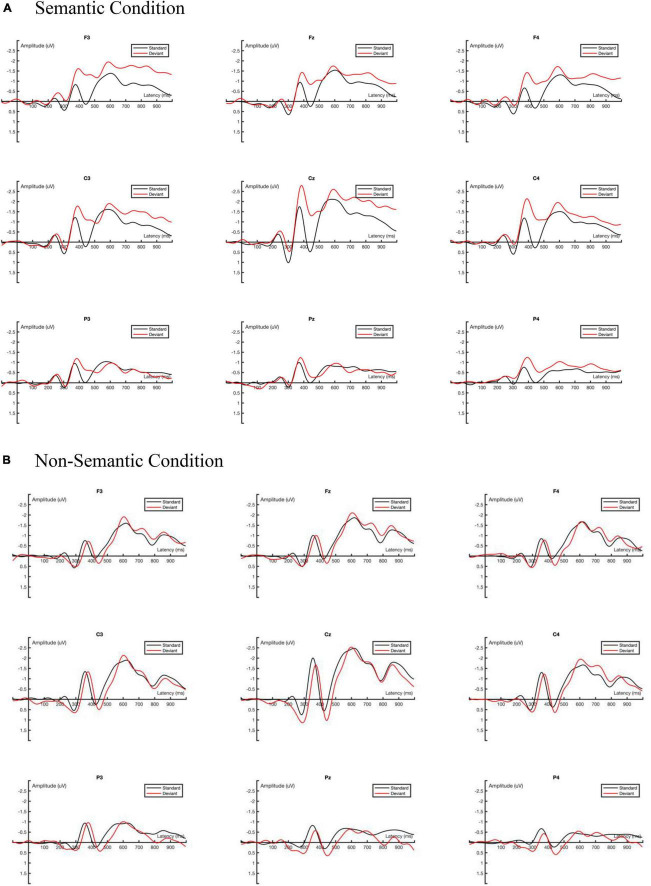
Ground averaged ERP waveforms. **(A)** The ground averaged waveforms of the semantic condition of nine interested channels. **(B)** The ground averaged waveforms of the non-semantic condition of nine interested channels.

The average ERP amplitude was first analyzed using three-factor repeated measurement ANOVA with two experimental conditions (semantic vs. non-semantic) × two stimulus types (standard vs. deviant) × nine electrodes (F3, Fz, F4, C3, Cz, C4, P3, Pz, P4). Greenhouse-Geisser method was used to adjust the lack of sphericity (Abdi, 2010). The ground-averaged waveforms generated by the standard and deviant stimuli under different experimental conditions (semantic vs. non-semantic) are shown in Figure 4.

In the time window of 370–450 ms, the main effect of the experimental conditions was significant, *F*_(1, 16)_ = 14.554, *p* = 0.002, η_p_^2^ = 0.476. The mean amplitude of the semantic condition (−0.937 μV) was significantly more negative than that of the non-semantic condition (−0.158 μV).

The main effect of the stimulus type was significant *F*_(1, 16)_ = 6.904, *p* = 0.018, η_p_^2^ = 0.301. The mean amplitude of the standard stimulus (−0.232 μV) was significantly less negative than that of the deviant stimulus (−0.762 μV).

The interaction effect of the experimental condition × stimulus type was significant, *F*_(1, 16)_ = 7.611, *p* = 0.014, η_p_^2^ = 0.322. The simple effect analysis results suggested that in the semantic condition, the mean amplitude of the standard stimulus (−0.352 μV) was significantly less negative than that of the deviant stimulus (−1.321 μV), *p* = 0.002.

The interaction effect of electrode × stimulus type was significant, *F*_(8, 128)_ = 2.847, *p* = 0.043, η_p_^2^ = 0.151. The simple effect analysis results indicated that in the F3, F4, C3, Cz, and C4 channels, the mean amplitude of the standard stimulus was significantly less negative than that of the deviant stimulus, *p* < 0.05.

### 4.2 Analysis of MMN

**FIGURE 5:**
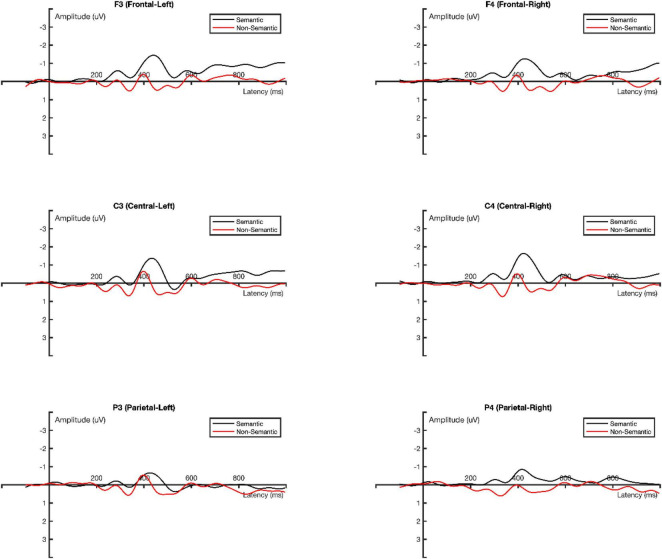
Ground Averaged MMN waveforms.

The average amplitude of MMN obtained from the deviant stimulus minus the standard stimulus was analyzed using three-factor repeated measures ANOVA: two experimental conditions (semantic vs. non-semantic) × 3 scalp distribution types (frontal, central, and parietal area) × 2 hemispherical positions (left vs. right). Greenhouse-Geisser method was used to adjust the lack of sphericity (Abdi, 2010). The ground-averaged MMN waveforms for the two experimental conditions (semantic vs. non-semantic) are shown in Figure 5.

In the time window of 370–450 ms, the main effect of the experimental conditions was significant, *F*_(1, 16)_ = 8.040, *p* = 0.012, η_p_^2^ = 0.334. The mean amplitude of MMN in the semantic condition (−0.974 μV) was significantly more negative than that in the non-semantic condition (−0.101 μV).

**FIGURE 6:**
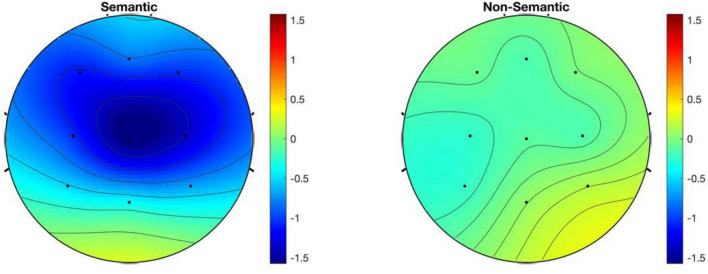
The distribution maps of MMN mean amplitude in different conditions.

The main effect of the scalp distribution type was marginally significant, *F*_(2, 32)_ = 3.360, *p* = 0.072, η_p_^2^ = 0.174 (Figure 6). The pairwise comparison results indicated that the mean amplitude of MMN in the central area (−0.711 μV) was significantly more negative than that in the parietal area (−0.330 μV), *p* = 0.003. Additionally, the mean amplitude of MMN in the left hemisphere (−0.559 μV) was more negative than that in the right hemisphere (−0.516 μV).

## 5 Discussion

The statistical results indicated that there was a significant difference between the mean amplitude in the semantic condition and that in the non-semantic condition, thereby illustrating that the brain processes the tone two (mid-rising) interrogative word “shui” differently when with the lexical semantics and when without the lexical semantics (Hum version). As the experiment used the passive oddball paradigm and the MMN reflects automatic processing at the pre-attentive level, the brain could distinguish the lexical semantics of the tone two interrogative word.

Additionally, the simple effect analysis results of the interaction effect on the experimental condition (semantic vs. non-semantic) and stimulus type (standard vs. deviant) suggested that the deviant stimulus (interrogative intonation) could only evoke MMN in the semantic condition. This result means that when the tone two interrogative word “shui” contains the lexical semantics, the brain can distinguish between the declarative and interrogative intonations. However, when there is only the acoustic stimulus (hum version “shui”), the brain cannot process the different intonation (declarative vs. interrogative) in tone two (mid-rising). These results were consistent with those of a study conducted by Ren et al. (2011). This might be because both the interrogative intonation and Mandarin tone two (mid-rising) words end with the mid-rising curve of their acoustic feature. The pre-attention level brain processing cannot differentiate between the interrogative intonation and tone two (mid-rising) endings, but the brain is also sensitive to the interrogative words in declarative intonation, and interrogative intonation might be because those words are always used for extra questions.

In addition, the simple effect analysis results of the interaction effect of electrodes × stimulus type illustrate that MMN only existed in the frontal (F3, F4) and central (C3, Cz, and C4) scalp areas, which means that at the pre-attention level, the domain brain regions that processed the intonation information were the frontal and central areas. The findings show that lexical semantic function is important in intonation processing at the pre-attention level, and it dominates processing in the frontal and central areas.

In this study, the time window of MMN initiation ranged from 370 to 450 ms, which is later than the usual time window of MMN generation (Näätänen et al., 2007; Winkler, 2007). This might be due to the particularity of the selection of experimental materials. In previous studies, researchers mostly used monosyllabic words for research, but the tone two (mid-rising) interrogative word “shui” contains the fricative sound “sh,” and “shui” is a multi-syllable word. From the contour curve of the sound, it can be observed that the real pitch is produced after the “sh” sound, which may result in the delay of the MMN time window owing to the different syllables of the Chinese character itself and the position where the meaning is generated. Additionally, there is no clear evidence of the domain hemisphere when processing the intonation of declaratives and interrogatives, which supports the functional and comprehensive hypotheses that the processing of intonation is based on the function of language, and both brain regions are involved in the processing.

## 6 Conclusion

This study made the first endeavor to explore the intonation processing of tone two (mid-rising) interrogative words in Mandarin at the pre-attentive level. Using EEG recordings of MMN, we were able to provide novel evidence that lexical semantics strongly modulate pre-attentive brain responses to intonation contours. The significant differences in MMN amplitude between semantic and non-semantic stimuli demonstrate that the brain rapidly distinguishes between interrogative and declarative intonations only when words carry lexical semantic information. This finding supports a functional account of early intonation processing, rather than an acoustic account, which is consistent with the view that the brain processes speech prosody in relation to linguistic function. The results of this study indicate that frontal and central cortical regions underlie automatic intonation processing, thereby elucidating the neural generators and temporal dynamics involved. Overall, the data demonstrated that language experience shapes pre-attentive auditory processing to be maximally sensitive to linguistically relevant pitch patterns in speech. The passive oddball paradigm with Mandarin tone two (mid-rising) interrogatives provides an elegant way to probe the early functional processing of intonation before attention or awareness. Therefore, this study contributes to the understanding of the early neural processing of linguistic intonation. However, the experiments were conducted using a single word–“shui,” meaning “who” (“谁”), and only assessed with the second tone. Future studies should explore different tones, using other interrogative Mandarin words, to study intonation processing.

## Data availability statement

The raw data supporting the conclusions of this article will be made available by the authors, without undue reservation.

## Ethics statement

The requirement of ethical approval was waived by the School of Educational Science and Technology, Anshan Normal University for the studies involving humans because of the regulations of the School of Educational Science and Technology, Anshan Normal University. The studies were conducted in accordance with the local legislation and institutional requirements. The participants provided their written informed consent to participate in this study.

## Author contributions

RW: Formal analysis, Investigation, Methodology, Software, Validation, Writing – original draft. MW: Conceptualization, Data curation, Resources, Visualization, Writing – original draft. GG: Supervision, Writing – review and editing.

## Footnotes

1
http://www.praat.org

2
https://www.egi.eu/
